# The association of early-life exposure to ambient PM_2.5_ and later-childhood height-for-age in India: an observational study

**DOI:** 10.1186/s12940-019-0501-7

**Published:** 2019-07-09

**Authors:** Dean Spears, Sagnik Dey, Sourangsu Chowdhury, Noah Scovronick, Sangita Vyas, Joshua Apte

**Affiliations:** 1 Department of Economics and Population Research Center, University of Texas at Austin 2225 Speedway, Austin, TX 78712 USA; 2 Economics and Planning Unit, Indian Statistical Institute, Delhi Centre, Delhi, 110016 India; 3 Centre for Atmospheric Sciences, Indian Institute of Technology Delhi, Hauz Khas, New Delhi, 110016 India; 4 Centre of Excellence for Research on Clean Air, Indian Institute of Technology Delhi, Hauz Khas, New Delhi, 110016 India; 5 Rollins School of Public Health, Emory University, Atlanta, USA; 6 Department of Civil, Architectural and Environmental Engineering, University of Texas at Austin, Austin, USA

**Keywords:** Child height, Ambient air pollution, PM_2.5_, India

## Abstract

**Background:**

Children in India are exposed to high levels of ambient fine particulate matter (PM_2.5_). However, population-level evidence of associations with adverse health outcomes from within the country is limited. The aim of our study is to estimate the association of early-life exposure to ambient PM_2.5_ with child health outcomes (height-for-age) in India.

**Methods:**

We linked nationally-representative anthropometric data from India’s 2015–2016 Demographic and Health Survey (*n* = 218,152 children under five across 640 districts of India) with satellite-based PM_2.5_ exposure (concentration) data. We then applied fixed effects regression to assess the association between early-life ambient PM_2.5_ and subsequent height-for-age, analyzing whether deviations in air pollution from the seasonal average for a particular place are associated with deviations in child height from the average for that season in that place, controlling for trends over time, temperature, and birth, mother, and household characteristics. We also explored the timing of exposure and potential non-linearities in the concentration-response relationship.

**Results:**

Children in the sample were exposed to an average of 55 *μ* g/m^3^ of PM_2.5_ in their birth month. After controlling for potential confounders, a 100 μg/m^3^ increase in PM_2.5_ in the month of birth was associated with a 0.05 [0.01–0.09] standard deviation reduction in child height. For an average 5 year old girl, this represents a height deficit of 0.24 [0.05–0.43] cm. We also found that exposure to PM_2.5_ in the last trimester in utero and in the first few months of life are significantly (*p* < 0.05) associated with child height deficits. We did not observe a decreasing marginal risk at high levels of exposure.

**Conclusions:**

India experiences some of the worst air pollution in the world. To our knowledge, this is the first study to estimate the association of early-life exposure to ambient PM_2.5_ on child height-for-age at the range of ambient pollution exposures observed in India. Because average exposure to ambient PM_2.5_ is high in India, where child height-for-age is a critical challenge in human development, our results highlight ambient air pollution as a public health policy priority.

**Electronic supplementary material:**

The online version of this article (10.1186/s12940-019-0501-7) contains supplementary material, which is available to authorized users.

## Background

India experiences some of the worst particulate air pollution in the world, with mean PM_2.5_ concentrations consistenly above World Health Organization guidelines [1–3]. Due to these high exposures, the Disease Burden of India study recently estimated that 12.9 (11.4–14.4) million disability adjusted life years (DALY) and 149.8 (132.3–167.6) thousand deaths annually were attributable to PM_2.5_ in children under 5 years of age [4]. The child mortality burden due to household PM_2.5_ exposure in India is also large, estimated at 50 (30–60) thousand deaths per year. These estimates, however, rely on concentration-response functions that were developed from epidemiological studies carried out primarily in high-income countries. Moreover, the empidemiological studies used for constructing these estimates do not consider impacts on a number of child health outcomes that have been linked to PM_2.5_ exposure, including sudden infant death syndrome [5], low birth weight [6, 7], intrauterine growth retardation [8] and reduced size [9].

Unlike many developed countries, India does not have a vital registration system, making it difficult to study mortality, a commonly used outcome variable in the air pollution literature. Another widely-studied marker of early-life health insults is the average height of children. Children in India are unusually short compared to international standards [10]. Many causes of this child height deficit have been proposed in the demographic, epidemiological, and econometric literatures, including poor sanitation and maternal nutrition [11, 12]. Exposure to PM_2.5_ from household solid fuel use for cooking and heating has also been associated with child growth in India [13, 14], but to our knowledge, no study from India has explored the link between ambient air pollution and child growth. One study from Bangladesh [8] observed that the risk of child stunting and wasting was positively associated with higher levels of in utero exposure.

In this study, we examine the association of in utero and early-age ambient PM_2.5_ exposure on child height-for-age in India. We do so in a representative sample of Indian children – a population exposed to a large range of ambient PM_2.5_ – using data from the 2015–2016 Demographic and Health Survey (DHS), matched to air pollution data, as measured by satellite remote sensing. The association between child height-for-age and early-life exposure to air pollution is estimated using an approach that accounts for fixed differences across villages, secular trends over time, and district-specific seasonal patterns. We also investigate the shape of the concentration-response function.

## Data and methods

### India’s 2015–2016 demographic and health survey

Data on child height and potential confounders are taken from India’s most recent DHS survey (note that in India the DHS is also known as the National Family Health Survey). These data were collected from a nationally-representative sample of women of reproductive age. The survey visited all 640 Indian districts that existed at the time of the 2011 Census, and was designed to be representative at the district level. These data were collected between January 2015 and November 2016.

In our analysis, the outcome (dependent) variable is a child’s height-for-age *z*-score, scaled according to the World Health Organization 2006 reference population mean and standard deviation by sex and age-in-months [15]. In the DHS, height is measured for children less than 5 years old at the time of the survey. The sex and month of birth (e.g. August 2011) is also recorded for each child with measured height.

### Air pollution data by district-month

Each child was assigned the average ambient PM_2.5_ exposure in his or her district of residence during the month in which he or she was born. This matching assumes that the district where children live at the time of the survey is the same as the district where children lived when they were born.

Because India lacks ground-based PM_2.5_ measurements at a spatial resolution sufficient for our study design, we used satellite-derived PM_2.5_. Specifically, we use the Multiangle Imaging SpecroRadiometer (MISR) retrieved daily aerosol optical depth (AOD) V22 product at 17.6 km × 17.6 km spatial resolution to estimate PM_2.5_ with the help of a spatially and temporally varying conversion factor (ƞ). ƞ is derived from GEOS-Chem chemical transport model simulations and depends on aerosol vertical distribution, emissions, and meteorological factors like temperature, relative humidity, and precipitation. Details about the conversion factor ƞ are discussed elsewhere [2, 16, 17]. The MISR AOD product was previously and extensively evaluated for the Indian subcontinent [18]. The satellite-retrieved PM_2.5_ was bias-corrected using coincident ground-based quality controlled measurements following our earlier study and has ~ 10% uncertainty [2, 19]. The district-level statistics are extracted using the shape files of the district boundaries in ArcGIS. We generated a monthly PM_2.5_ exposure database for 15 years (2001–2015), although because height is only measured in the DHS for children under five, no child in our sample was born before 2010.

### Temperature data by district-month

Considering the large spatio-temporal heterogeneity in temperature across India [20], we control for temperature in the month and district of birth. Monthly temperature data at the 0.125° × 0.125° (approximately, a 12 km × 12 km grid) resolution was obtained from the European Centre for Medium Range Weather Forecast (ECMWF) ERA-INTERIM dataset. Mean district-level temperature was estimated using a spatially weighted average of the 0.125° × 0.125° grid cells in the district.

### Main statistical approach

The central empirical strategy of this paper is fixed effects regression, with child height-for-age as the outcome (dependent) variable, and early-life district-month exposure to ambient PM_2.5_ as the independent variable (exposure) of interest. Fixed effects regression has been identified as a useful tool in epidemiological analyses to control for unobserved characteristics that are common across observations within groups, time periods, or individuals [21], and has been successfully applied in prior epidemiological studies of air pollution-health relationships [8]. In our analysis, we include fixed effects for birth place, seasonal patterns in the district, and year. We therefore study whether deviations in seasonal average PM_2.5_ in a particular place (village or urban block) are associated with deviations in child height from the average for that same season in that same place. As a result, our study asks the question: is exposure to PM_2.5_ in the month of birth that is higher than the seasonal average associated with heights that are shorter than average for that place and season of birth?

In India, and other countries where environmental risks are widespread, the average height-for-age z-score declines in the first 2 years of life, reflecting the accumulating impact of early-life health insults on a child’s growth [22]. Because age is predictably correlated with height-for-age, each regression also controls for 119 age-in-months-by-sex indicators, one for each age in months from zero to 59, for girls and boys separately, and excluding one to avoid perfect multicollinearity. This type of adjustment is standard in the literature on child height [10, 11].

Our main models take the following form:

1 $$ {h}_{ipdmy}=\beta {x}_{dm y}+{\mu}_1{t}_{dm y}+{\mu}_2{t}_{dm y}^2+\rho\ {momh}_{ipdmy}+{\boldsymbol{\alpha}}_{pd}+{\boldsymbol{\gamma}}_{dm}+{\boldsymbol{\delta}}_y+{\boldsymbol{X}}_{ipdmy}\boldsymbol{\theta} +{\varepsilon}_{ipdmy} $$

where *i* indexes individual children, *p* places (survey primary sampling units – PSUs – such as urban blocks or rural villages), *d* districts*, m* calendar month of birth (such as February), and *y* calendar year of birth (such as 2012). The dependent variable, *h*, is child *i*’s height-for-age *z*-score. The independent variable of interest, *x*_*dmy*_, is PM_2.5_ in district *d* in month *m* of year *y*, corresponding to child *i*’s birth month. Similarly, *t*_*dmy*_ is temperature in that same district-month. We include temperature as a quadratic in order to allow for nonlinearities in its association with child height. *momh*_*ipdmy*_ is the height of the mother’s child, in centimeters, a proxy for the health and socioeconomic status of the mother. Fixed effects are ***α***_*pd*_, 27,266 local places (PSUs); ***γ***_*dm*_, 7679 categories of district-month (such as for Februarys in Sitapur district, or Aprils in Kanpur district); and ***δ***_*y*_, 6 calendar years, to capture any secular time trend. Child-level covariates ***X***_*ipdmy*_ include age-by-sex fixed effects and other covariates that have been associated with child height. These include birth characteristics (mother’s age at birth [23], birth order [24], whether the delivery occurred in a hospital or health facility [25], and whether it was a multiple birth [23]), mother characteristics (whether she smokes [23], the total number of children born to her by the time of the survey [24], and her relationship to the household head [26]), and household-level covariates (caste [12], religion [27], solid fuel use [13], open defecation [11], and drinking water source [8]).

This statistical strategy was designed to address several potential sources of confounding. The strategy allows us to add fixed effects and covariates in stages to verify that the main effect estimate, $\hat{\beta}$, is robust to respecification. In particular, we first estimate the model without PSU fixed effects. PSU fixed effects would account for any fixed geographic differences in factors known to affect child height, such as the presence and quality of markets [28], local open defecation [29], or the religious composition of the neighborhood [30]. We then add birth, mother, and household characteristics, as described above, which are intended to control for other known determinants of child height. While birth-level covariates reflect characteristics at the time of birth, mother and household level characteristics are observed at the time of the survey. Many of these characteristics, such as mother’s height, caste, and religion, are not likely to have changed over time. However, other characteristics, such as mother smoking, mother’s relationship to the household head, and water source may have changed. The use of solid fuels for cooking and open defecation has been changing relatively slowly in India over time [12, 31], and so these variables are likely to be highly correlated with household behaviors at the time of the child’s birth, even though they are only observed at the time of the survey. Our a priori preferred specification, however, includes birth characteristics only because these variables reflect the environment at the time of birth, and not at the time of the survey. All subsequent analyses build off of this preferred specification.

All of our main model specifications control for district-month fixed effects, a tool which has been used in the literature to control for seasonal trends [32]. This strategy allows each district to have any distinct seasonal pattern, and identifies effects off of deviations from each district’s seasonal patterns. Controlling for seasonal trends is important because pollution in India is highly seasonal [33], and later life outcomes such as educational attainment are also known to be predicted by seasonal patterns [34]. Since child height is also correlated with these outcomes [35], season is likely to be a confounder. A month fixed effect by itself would control for seasonality that is common across all of India. However, since pollution patterns are highly localized, we include district-month fixed effects, which allow seasonal patterns to be different in each district, and therefore we control for *local* seasonality. We also include a sensitivity check with finer (PSU-month) controls for seasonality.

Finally, we conduct a falsification test, in which we control for ambient PM_2.5_ in the same district-month, but 2 years before the month of birth; if our identification strategy is credible, this control should not predict height nor change our estimate. Standard errors are clustered by 640 districts, to permit arbitrary correlation of error terms over space and time within districts [36].

DHS data include sampling weights, to be used to generate estimates that are representative of the population of Indian children under five. Although we use weights for our summary statistics in Table 1, sampling weights are not appropriate for estimating associations [37], so we do not use them in any of our statistical models. In Additional file 1: Table S1, we also calculate summary statistics without sampling weights, which are very similar to the statistics presented in Table 1.

**Table 1:** Summary statistics describing sample of children with measured height from India’s 2015–16 DHS, reported for the full sample and by PM_2.5_ quintiles in the month and district of birth

	full sample	PM_2.5_ quintile				
		1	2	3	4	5
PM_2.5_ in birth month, *μ* g/m^3^	54.9	15.3	30.1	45.7	65.2	118.2
height-for-age z-score	−1.50	−1.35	−1.45	−1.52	− 1.59	−1.60
temperature (Celsius)	16.5	17.5	17.0	17.2	16.9	13.7
age in months	30.7	31.9	31.2	30.7	30.4	29.1
girls	0.48	0.49	0.48	0.48	0.47	0.48
mother’s age at birth	24.3	24.2	24.1	24.2	24.4	24.5
birth order	2.18	1.97	2.09	2.18	2.30	2.38
institutional delivery	0.79	0.86	0.83	0.80	0.75	0.72
mother’s height (cm)	151.7	152.2	151.9	151.6	151.4	151.2
# of children born to mother	2.46	2.23	2.37	2.46	2.59	2.67
mother smokes	0.06	0.07	0.06	0.06	0.06	0.05
rural	0.72	0.67	0.71	0.72	0.74	0.76
uses solid fuels for cooking	0.63	0.53	0.60	0.63	0.68	0.72
defecates in open	0.47	0.40	0.47	0.49	0.51	0.49
born at residence	0.91	0.86	0.90	0.91	0.92	0.94
n (children under 60 months)	218,152	52,947	43,942	40,831	40,551	39,881

*Note:* Each number, other than sample sizes in the bottom row, is a sample mean. Girl, institutional delivery, mother smokes, rural, uses solid fuels for cooking, defecates in open, and born at residence are each indicators (1 or 0) for that property of the child, mother or household. Sample means and quintiles are computed with DHS sampling weights (which is why *n* is not constant across quintiles)

### Age of exposure

Our primary goal in this study is to investigate the effect on child height of exposure to ambient PM_2.5_ in the month of birth. The most vulnerable period of exposure is an active area of research [38, 39], but we chose exposure in the month of birth a priori because it represents an important period for early-life development [40]. Nevertheless, in additional analyses we also consider exposure at other ages by averaging over three-month periods, from − 8 to − 6 months before birth (approximately the first trimester of pregnancy) to 10 to 12 months after birth (approximately the last quarter of the first year of life). Average PM_2.5_ in each age of exposure is used as the independent variable in a separate regression:

2 $$ {h}_{ipdmy}=\beta \left(\frac{x_{dm y}^{+0}+{x}_{dm y}^{+1}+{x}_{dm y}^{+2}}{3}\right)+{\mu}_1{t}_{dm y}+{\mu}_2{t}_{dm y}^2+\rho\ {momh}_{ipdmy}+{\boldsymbol{\alpha}}_{pd}+{\boldsymbol{\gamma}}_{dm}+{\boldsymbol{\delta}}_y+{\boldsymbol{X}}_{ipdmy}\boldsymbol{\theta} +{\varepsilon}_{ipdmy} $$

where indices and fixed effects are as in regression eq. (1), but the covariates ***X*** include only the age-in-months-by-sex indicators and birth characteristics, as these variables reflect attributes at the time of birth, and not at the time of the survey. Therefore, this model builds off of the preferred specification discussed in the previous section.

### Shape of the concentration-response function

The shape of the concentration-response function has been a focus of the air pollution literature, in light of its importance for policy responses [41]. Although the prior literature has suggested the possibility of diminishing marginal risks at higher levels of exposure, there is little well-identified evidence on exposure to PM_2.5_ at levels as high as in India during the period studied, or for child height as the outcome [42, 43]. Therefore, we perform three analyses in which we allow the shape of the concentration-response function to differ from the linear form in eq. (1):

3 $$ {h}_{ipdmy}=\beta f\left({x}_{dm y}\right)+{\mu}_1{t}_{dm y}+{\mu}_2{t}_{dm y}^2+\rho\ {momh}_{ipdmy}+{\boldsymbol{\alpha}}_{pd}+{\boldsymbol{\gamma}}_{dm}+{\boldsymbol{\delta}}_y+{\boldsymbol{X}}_{ipdmy}\boldsymbol{\theta} +{\varepsilon}_{ipdmy} $$

First, we substitute in the natural log of PM_2.5_ in one specification, and a linear spline at the median of PM_2.5_ in another. Then we allow polynomial shapes of the concentration-response curve, of degree 1 through 5. Finally, we implement a Box-Cox power transformation of the form *f*(*x*) = *x*^*λ*^, for coefficients *λ* in steps of 0.1 from 0.1 to 2.0. We implement each power transformation in a separate model, and plot the resulting log-likelihoods. If likelihood is maximized near *λ* = 1, then this procedure would suggest that a linear concentration-response function best fits the data. As in the age of exposure analysis, the covariates ***X*** include only the age-in-months-by-sex indicators and birth characteristics, as these variables represent attributes at the time of birth, and not at the time of the survey.

### Informed consent

Because we study publicly-available, anonymized data on child height, our study is classified as “not human subjects research” and informed consent is not required.

All analyses in the paper were computed with Stata 12.1.

## Results

### Summary and descriptive statistics

**Fig. 1:**
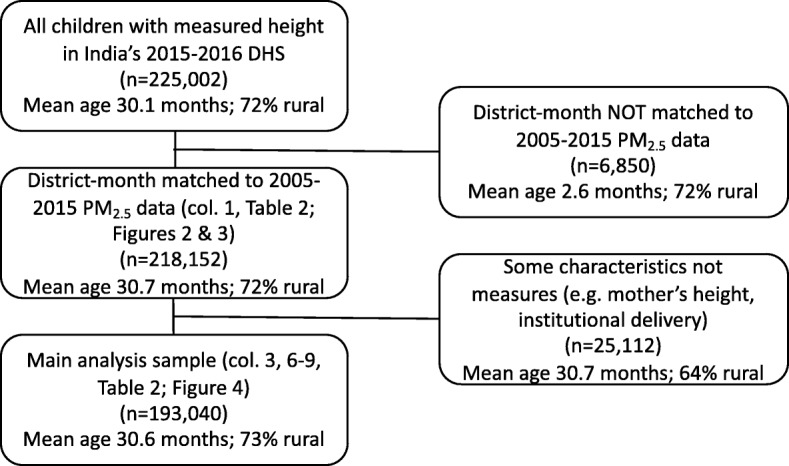
Study sample with excluded or missing observations. Note: In Table 2, some samples are smaller than 193,040 because the regression models ignore categories within which there is no variation in the independent variable

Height was measured for 225,002 children under five in the DHS. We were able to match air pollution data to children born from February 2010 to December 2015, who are 97% of those with measured height, resulting in a final sample of 218,152 children (Fig. 1). Summary statistics as sample means for these children are presented in Table 1, reported for the full sample, as well as by quintiles of ambient PM_2.5_ exposure. Across the whole sample, children were exposed to an average of 55 *μ* g/m^3^ in their month of birth, although with substantial variation. 92% of children were born while the mother was living in her current residence. The results also highlight that children who are exposed to higher ambient PM_2.5_ tend to be disadvantaged in other ways: they come from larger families, have shorter mothers, live in households that are more likely to defecate in the open, and use solid fuels for cooking.

**Fig. 2:**
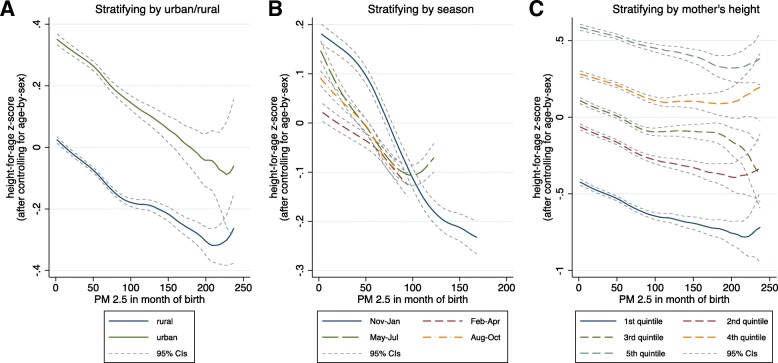
Crude associations between child height and exposure to PM_2.5_ in the month of birth. Note: Panel **a** stratifies by urban/rural, Panel **b** by season of birth, and Panel **c** by mother’s height. Curves are kernel-weighted local regressions. The vertical axis in all panels is the residual of child height-for-age, after controlling for age-in-months by sex indicators. In Panel B, observations of PM_2.5_ that are greater than the 95th percentile for each season are left out because a sufficient number of observations are not available to construct means for pollution levels that are very high for the season

Location [44, 45], time of year [33], and mother’s height, as seen in Table 1, are correlated with air pollution concentrations. Therefore, Fig. 2 plots crude associations between pollution and height, stratified by rural/urban, season, and mother’s height. Each panel in Fig. 2 presents locally-weighted kernel regressions of the relationship between ambient PM_2.5_ in the district-month of birth and height-for-age z-score residuals (after controlling only for age-by-sex, see methods for more discussion). We do not control for any other covariates in this figure. Panel A reveals a negative (downward) gradient – which is approximately linear - between ambient PM_2.5_ exposure and child height for both rural and urban children. Although the range of PM_2.5_ exposure is similar in both rural and urban areas, the former are shorter, on average, because they are more exposed to other factors associated with growth faltering [12, 29]. Panel B demonstrates that ambient PM_2.5_ reaches the highest levels in winter (November through January), and that a similar downward gradient is present in all seasons. Panel C suggests that the association is not driven by mother’s height, since the downward gradient exists for each quintile of mother’s height.

### Exposure in the month-of-birth

Table 2 presents our main results: fixed effects regression results following Eq. 1. For ease of interpretation, results are presented for a 100 *μ* g/m^3^ increase in PM_2.5_. Column 1 shows coefficients from a regression that includes age-by-sex fixed effects, district-month fixed effects, and year of birth fixed effects. Column 2 adds PSU fixed effects, temperature, and mother’s height. Columns 3, 4, and 5 progressively add birth, mother, and household characteristics, respectively. Across the alternative specifications in columns 1 through 5, a 100 *μ* g/m^3^ increase in ambient PM_2.5_ exposure is associated with an approximately 0.05 standard deviation decrease in child height-for-age z-score. Because columns 4 and 5 include covariates that are measured at the time of the survey, and do not necessarily reflect the environment at the time of birth, Column 3 represents our a priori preferred specification. Column 6, reports the falsification test: as expected, ambient PM_2.5_ exposure 2 years before the child is born does not predict height and does not change the coefficient of interest. Columns 7 and 8 are tests of the linearity assumption and are discussed further below.

**Table 2:** Association of district-level PM_2.5_ (per 100 μg/m^3^) in month of birth with child height-for-age z-score

	(1)	(2)	(3)	(4)	(5)	(6)	(7)	(8)
PM_2.5_ ÷ 100	−0.0546**	−0.0500*	− 0.0491*	−0.0486*	− 0.0428+	−0.0525*		−0.0216
	(0.0201)	(0.0218)	(0.0220)	(0.0220)	(0.0222)	(0.0231)		(0.0574)
PM_2.5_ ÷ 100						−0.0149		
24 months earlier						(0.0226)		
ln(PM_2.5_)							−0.0175+	
							(0.0104)	
PM_2.5_ ÷ 100								−0.0370
above median spline								(0.0694)
n (children under 60 months)	218,152	192,771	192,303	192,302	182,079	192,303	192,303	192,303
age in months × sex FEs	yes	yes	yes	yes	yes	yes	yes	yes
district-month FEs	yes	yes	yes	yes	yes	yes	yes	yes
year of birth FEs	yes	yes	yes	yes	yes	yes	yes	yes
PSU FEs		yes	yes	yes	yes	yes	yes	yes
mother’s height (cm)		yes	yes	yes	yes	yes	yes	yes
temperature & temperature^2^		yes	yes	yes	yes	yes	yes	yes
birth characteristics			yes	yes	yes	yes	yes	yes
mother characteristics				yes	yes			
household characteristics					yes			

*Note:* All columns present ordinary least squares fixed effects regressions with the child’s height-for-age *z*-score as the dependent variable. *FE* fixed effect, *PSU* primary sampling unit (urban block or rural village). Standard errors clustered by 640 districts in parentheses. + *p* < 0.10; * *p* < 0.05; ** *p* < 0.01. In column 8, the spline variable is zero below the median PM_2.5_ and is identical to PM_2.5_ above the median. Sample sizes vary because some fixed effects categories lack within-category variation in the independent variable (resulting in that category being dropped), and because not all children’s mothers’ heights were measured. Birth characteristics include mother’s age at birth, birth order, whether the delivery occurred in a hospital or health facility, and whether it was a multiple birth. Mother characteristics include whether she smokes, the total number of children born to her by the time of the survey, and her relationship to the household head. Household-level characteristics include caste, religion, solid fuel use, open defecation, and drinking water source

### Age of exposure

**Fig. 3:**
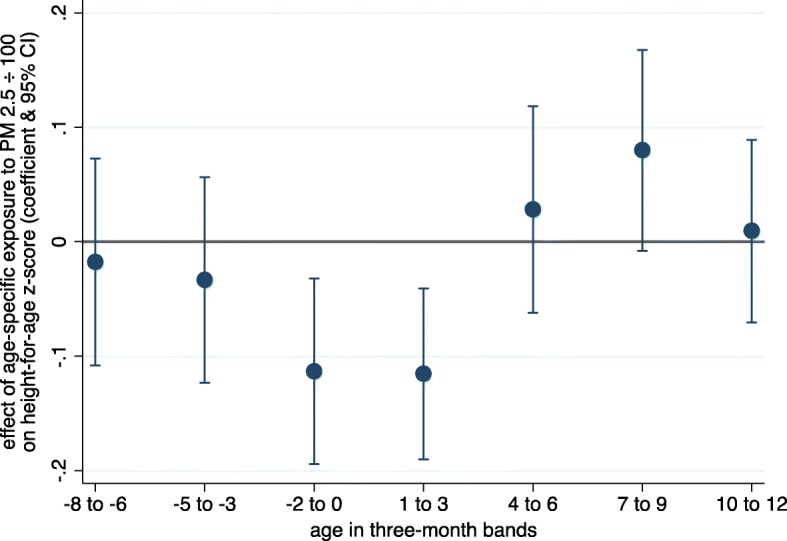
Effects of PM_2.5_ exposure at various ages. Note: Dots denote point estimates and lines denote 95% confidence intervals. Each result shown is from a separate fixed effects regression of child height-for-age on the average exposure to PM_2.5_ in the months, relative to birth, specified along the horizontal axis

Figure 3 presents the association of PM_2.5_ and child growth given different time periods of exposure (see Eq. 2 above for modeling details). Of the seven time periods explored, two show significant (*p* < 0.05) adverse effects on child growth – the last trimester in utero and the period just after birth. No other periods of exposure are significantly associated with child height. These results are consistent with evidence in the literature that shocks in utero and early-life are critical for child development outcomes [40, 46].

### Shape of concentration-response function

The three tests for non-linear concentration-response functions each failed to reject that a linear shape best fits the data. Moreover, each approach suggests that, if anything, effects may be steeper at higher concentration levels. Specifically, column 7 of Table 2 shows that a natural log functional form – consistent with a concentration-response function exhibiting diminishing marginal costs – fits the data less well than the linear form. Column 8 includes a linear spline that allows a different slope above the median level of ambient PM_2.5_; although the two PM_2.5_ terms are jointly statistically significant at the 10% level (*F* = 2.72; *p* = 0.067), neither is individually significantly different from zero. Although this model does not fit the data better than a simple linear form, the negative sign on the coefficient suggests the possibility of a steeper concentration-response function at higher levels of exposure. Additional file 1: Figure S1 demonstrates that none of the polynomial forms we tested (quadratic through quantic) improve on a linear functional form, while Additional file 1: Figure S2 – the Box-Cox transformation – indicates that a model with slightly increasing marginal effects may best fit the data.

In Additional file 1: Table S2, we present results from statistical analyses similar to columns 6 through 8 of the the main Table 2, the difference is that models presented in the supplementary table include all coverates, including birth, mother, and household characteristics, rather than birth characteristics only. The inclusion of these additional control variables does not change the interpretation of these analyses. We also show that the model is robust to replacing district-month fixed effects with PSU-month fixed effects, a finer measure of seasonality.

## Discussion

We report the first evidence of an association between ambient PM_2.5_ exposure and child height in India by using the country’s most recent DHS, which measures children under 5 years old in a nationally representative sample of reproductive age women. We find that an increase in PM_2.5_ of 100 *μ* g/m^3^ in the month of birth is associated with a decrease of 0.05 height-for-age standard deviations; for an average 5 year old girl, this would equate to a height deficit of 0.24 cm. Consistent with evidence in the literature that shocks in utero and early-life are critical for child development outcomes [40, 46], we find evidence that exposure to PM_2.5_ during the last few months in utero and the first few months of life are associated with height deficits.

The average child in our data is exposed to a PM_2.5_ concentration of 55 *μ* g/m^3^ in her month of birth. Using the estimates from our analysis, this means that the average child is about 0.027 height-for-age standard deviations shorter than she would be if exposed to very low levels of air pollution at birth. For an average 5 year old girl, this represents a height deficit of 0.13 cm. Although this effect is small relative to other environmental factors affecting child health, such as open defecation [11], it influences all of the almost 30 million births per year that occur in India.

Moreover, the difference between the children in our sample most exposed to PM_2.5_ (at the 95th percentile) and the children least exposed (at the 5th percentile) is 116 *μ* g/m^3^. Therefore, based on our findings, the most exposed children in India are about 0.06 height-for-age standard deviations shorter than they counterfactually would be if they were exposed only at the lowest levels in our sample. This projected difference — 0.06 height-for-age standard deviations — is of the same order of magnitude as other height differences that have received sustained attention in the literature on the demography of child height: it is about half as large as the well-studied India-Africa height gap [11], and is about one-tenth of the height gap between children of literate versus illiterate mothers. Since child growth is highly correlated with early-life mortality [47], the associations we observed in this study are suggestive of an association between PM_2.5_ exposure and early-life survival. In the data we use for this analysis, a district where children are 0.06 height-for-age standard deviations shorter would be expected, on average, to have an infant mortality that is larger by 5 infant deaths per 1000 live births: a large difference that is approximately equal to Canada’s overall infant mortality rate.

Although child height has traditionally been interpreted as a measure of “malnutrition,” it is increasingly recognized to reflect the totality of early-life health insults, including both net available nutrition and losses due to diseases. Our study does not allow us to observe disease directly; however, mechanisms in the literature are consistent with the association that we document. For example, exposure to particulate matter is associated with lower birth weight [6, 7], which is in turn linked to stature in childhood [48]. Similarly, exposure to ambient air pollution is associated with the incidence of pneumonia [49, 50]. Respiratory infections, like pneumonia, sometimes occur with fevers which can suppress the appetite, and reduce nutrient intake [51]. Moreover, infection and inflammation are metabolically demanding and may reallocate resources at the expense of growth [52].

This study has several limitations. One is the possibility of residual confounding. For example, we were not able to control for potential co-pollutants such as ozone or NO_2_, for which data is not available. In addition, some variables included in our models were measured at the time of the survey rather than at the time of the child’s birth, such as open defecation and household solid fuel use. However, we have no reason to believe that these practices would have changed for a large proportion of households. Similarly, we assumed that surveyed mothers delivered their children in the same district in which they were surveyed. This assumption seems sound considering that 92% of children were recorded as being born while the mother was living in her current residence, and because migration across districts is relatively rare. Finally, we rely on district-level measures of exposure derived from satellite data, thus raising the possibility of measurement error. However, assuming this error is random, the consequence would be attenuation towards the null, meaning that the true size of the effect of PM_2.5_ on child height may be larger than we observe here. In light of these limitations, we encourage additional research on this topic. If possible, this would include other study designs (cohort studies, natural experiments etc.) and, when available, finer-resolution estimates of exposure.

## Conclusions

To our knowledge, this is the first study to directly estimate the impact of early-life exposure to ambient PM_2.5_ on child height-for-age at the range of exposures found in India. Because average exposure to ambient PM_2.5_ is high in India, where child height-for-age is a critical challenge in human development, our results highlight ambient air pollution as public health policy priority. Ambient PM_2.5_ exposure is likely to increase in India in the near future [1, 53]. Therefore, the health burden that we quantify here could potentially increase unless appropriate policy action is taken to reduce air pollution throughout India. In particular, although policy conversations often focus on Delhi (and, to a lesser extent, other big cities), we find effects throughout India, and on both rural and urban children, suggesting that the policy challenges are broader than is commonly understood. Because child height has lasting consequences for human capital [10, 40], this is a problem with potential ramifications throughout the Indian society and economy.

## Additional file

Additional file 1: **Table S1.** Summary statistics describing sample of children with measured height from India’s 2015–16 DHS, computed without sampling weights. **Table S2.** Association of district-level PM_2.5_ (per 100 μg/m^3^) in month of birth with child height-for-age z-score with all covariates. **Figure S1.** Projected effects of PM_2.5_ on child height-for-age z-score, at increasing non-linearity. Each curve is the projected effect from a separate fixed effects regression where PM_2.5_ in the month of birth is specified as a polynomial of degree 1 through 5. *p*-values report joint *F* tests that all PM_2.5_ terms are zero. **Figure S2.** Box-Cox transformation of PM_2.5_ in month of birth: Each point plots the log likelihood of a separate fixed effects regression of PM_2.5_ transformed according to the coefficient on the horizontal axis. (DOCX 83 kb)

## Data Availability

The DHS 2015–2016 survey data for India are publicly available free of charge, and archived at https://www.dhsprogram.com/data/available-datasets.cfm. MISR AOD data used to generate the PM_2.5_ exposure are archived at https://misr.jpl.nasa.gov/getData/accessData/. The Stata do files used to arrive at the results depicted in this study are located at https://riceinstitute.org/wp-content/uploads/2019/07/hfa_PM25_public.txt.
