# Supplementary material for: The association of early-life exposure to ambient PM2.5 and later-childhood height-for-age in India: an observational study
Source: Environ Health. 2019 Jul 9;18:62. doi: 10.1186/s12940-019-0501-7 (PMC6617650; doi:10.1186/s12940-019-0501-7)
Supplement: Supplementary file 1 — Table S1. Summary statistics describing sample of children with measured height from India’s 2015–16 DHS, computed without sampling weights. Table S2. Association of district-level PM2.5 (per 100 μg/m3) in month of birth with child height-for-age z-score with all covariates. Figure S1. Projected effects of PM2.5 on child height-for-age z-score, at increasing non-linearity. Each curve is the projected effect from a separate fixed effects regression where PM2.5 in the month of birth is specified as a polynomial of degree 1 through 5. p-values report joint F tests that all PM2.5 terms are zero. Figure S2. Box-Cox transformation of PM2.5 in month of birth: Each point plots the log likelihood of a separate fixed effects regression of PM2.5 transformed according to the coefficient on the horizontal axis. (DOCX 83 kb) [file 12940_2019_501_MOESM1_ESM.docx]

Additional File 1

**Supplementary Table 1.** Summary statistics describing sample of children with measured height from India’s 2015-16 DHS, computed without sampling weights

|  |  | PM_2.5_ quantiles in the month and district of birth | | | | | |
| --- | --- | --- | --- | --- | --- | --- | --- |
|  | full sample | | 1 | 2 | 3 | 4 | 5 |
| PM_2.5_ in birth month | 51.5 | | 12.5 | 26.9 | 42.4 | 62.2 | 113.3 |
| height-for-age z-score | -1.51 | | -1.34 | -1.46 | -1.54 | -1.59 | -1.60 |
| temperature (Celsius) | 15.4 | | 15.4 | 15.3 | 15.9 | 16.2 | 14.2 |
| age in months | 30.9 | | 31.6 | 31.6 | 31.1 | 30.7 | 29.5 |
| girl | 0.48 | | 0.49 | 0.48 | 0.48 | 0.48 | 0.48 |
| mother's age at birth | 24.7 | | 25.1 | 24.6 | 24.5 | 24.5 | 24.6 |
| birth order | 2.27 | | 2.20 | 2.18 | 2.25 | 2.31 | 2.39 |
| institutional delivery | 0.76 | | 0.76 | 0.78 | 0.77 | 0.75 | 0.73 |
| mother's height (cm) | 151.7 | | 152.0 | 152.0 | 151.7 | 151.6 | 151.5 |
| # of children mother has | 2.56 | | 2.46 | 2.47 | 2.54 | 2.61 | 2.69 |
| mother smokes | 0.10 | | 0.19 | 0.10 | 0.09 | 0.07 | 0.05 |
| rural | 0.76 | | 0.74 | 0.76 | 0.77 | 0.77 | 0.77 |
| uses solid fuels for cooking | 0.68 | | 0.62 | 0.66 | 0.69 | 0.71 | 0.71 |
| defecates in open | 0.44 | | 0.31 | 0.44 | 0.50 | 0.50 | 0.48 |
| born at residence | 0.92 | | 0.89 | 0.92 | 0.92 | 0.93 | 0.94 |
| n (children under 60 months) | 218,152 | | 218,152 | 43,631 | 43,639 | 43,627 | 43,627 |

*Note:* Each number, other than sample sizes in the bottom row, is a sample mean. Girl, institutional delivery, mother smokes, rural, uses solid fuels for cooking, defecates in open, and born at residence are each indicators (1 or 0) for that property of the child or household.

**Supplementary Table 2.** Association of district-level PM_2.5_ (per 100 μg/m^3^) in month of birth with child height-for-age z-score with all covariates

|  | (1) | (2) | (3) | (4) |
| --- | --- | --- | --- | --- |
| PM_2.5_ ÷ 100 | -0.0469* |  | -0.00211 | -0.0905** |
|  | (0.0232) |  | (0.0581) | (0.0324) |
| PM_2.5_ ÷ 100 | -0.0182 |  |  |  |
| 24 months earlier | (0.0236) |  |  |  |
| ln(PM_2.5_) |  | -0.0140 |  |  |
|  |  | (0.0105) |  |  |
| PM_2.5_ ÷ 100 |  |  | -0.0548 |  |
| above median spline |  |  | (0.0700) |  |
| n (children under 60 months) | 182,079 | 182,079 | 182,079 | 94,123 |
| age in months × sex FEs | yes | yes | yes | yes |
| district-month FEs | yes | yes | yes |  |
| year of birth FEs | yes | yes | yes | yes |
| PSU FEs | yes | yes | yes |  |
| PSU-month FEs |  |  |  | yes |
| mother’s height (cm) | yes | yes | yes | yes |
| temperature & temperature^2^ | yes | yes | yes | yes |
| birth characteristics | yes | yes | yes | yes |
| mother characteristics | yes | yes | yes | yes |
| household characteristics | yes | yes | yes | yes |

*Note:* All columns present ordinary least squares fixed effects regressions with the child’s height-for-age *z*-score as the dependent variable. FE = fixed effect; PSU = primary sampling unit (urban block or rural village). Standard errors clustered by 640 districts in parentheses. + *p*  < 0.10; * *p* < 0.05; ** *p* < 0.01. In column 3, the spline variable is zero below the median PM_2.5_ and is identical to PM_2.5_ above the median. Sample sizes vary because some fixed effects categories lack within-category variation in the independent variable (resulting in that category being dropped), and because not all children’s mothers’ height was measured. Birth characteristics include mother’s age at birth, birth order, whether the delivery occurred in a hospital or health facility, and whether it was a multiple birth. Mother characteristics include whether she smokes, the total number of children born to her by the time of the survey, and her relationship to the household head. Household-level characteristics include caste, religion, solid fuel use, open defecation, and drinking water source.

**Supplementary Figure 1.** Projected effects of PM_2.5_ on child height-for-age z-score, at increasing non-linearity. Each curve is the projected effect from a separate fixed effects regression where PM_2.5_ in the month of birth is specified as a polynomial of degree 1 through 5. *p*-values report joint *F* tests that all PM_2.5_ terms are zero.

**Supplementary Figure 2.** Box-Cox transformation of PM_2.5_ in month of birth: Each point plots the log likelihood of a separate fixed effects regression of PM_2.5_ transformed according to the coefficient on the horizontal axis.
